# Pharmacometabolomics Identifies 3-Hydroxyadipic Acid, d-Galactose, Lysophosphatidylcholine (P-16:0), and Tetradecenoyl-l-Carnitine as Potential Predictive Indicators of Gemcitabine Efficacy in Pancreatic Cancer Patients

**DOI:** 10.3389/fonc.2019.01524

**Published:** 2020-01-29

**Authors:** Dongyuan Wu, Xinyuan Li, Xiaohan Zhang, Fang Han, Xin Lu, Lei Liu, Junsheng Zhang, Mei Dong, Huanjie Yang, Hui Li

**Affiliations:** ^1^Department of Biochemistry and Molecular Biology, Basic Medical Science College, Harbin Medical University, Harbin, China; ^2^Department of Pharmacy, Harbin Medical University Cancer Hospital, Harbin, China; ^3^School of Life Science and Technology, Harbin Institute of Technology, Harbin, China; ^4^College of Bioinformatics Science and Technology, Harbin Medical University, Harbin, China; ^5^College of Basic Medicine, Harbin Medical University, Harbin, China

**Keywords:** pancreatic carcinoma, metabonomics, chemotherapy, gemcitabine, predictive indicator

## Abstract

Gemcitabine (GEM)-based chemotherapy is the standard regimen for the treatment of pancreatic cancer (PC). However, chemoresistance is a major challenge in PC treatment. Reliable biomarkers are urgently needed to predict the response to GEM-based therapies. GEM-sensitive (GEM-S) and GEM-resistant (GEM-R) pancreatic carcinoma xenograft models were established, and GEM monotherapy and GEM plus nanoparticle albumin-bound paclitaxel (nab-PTX) doublet therapy were administered to GEM-S/R tumor-bearing mice. Metabolomic mass spectrometry (MS) analysis of serum, liver, and tumor samples was performed using an ultraperformance liquid chromatography-quadrupole time-of-flight mass spectrometer. The results showed that both GEM monotherapy and combination therapy significantly inhibited the tumor growth in GEM-S subgroup. However, in the GEM-R subgroup, tumor growth was not significantly inhibited by GEM monotherapy, but was significantly suppressed by GEM combination therapy. Metabolic profiling analysis by hierarchical cluster analysis and partial least squares discriminant analysis showed that the differences in metabolites were most significant in serum of three types of samples in the GEM-S/R subgroups, regardless of the administration of GEM monotherapy or combination therapy. The differential metabolite analysis of serum samples revealed 38 and 26 differential metabolites between the GEM-R and GEM-S subgroups treated with GEM monotherapy or combination therapy, and four common discriminating metabolites were investigated: 3-hydroxyadipic acid, d-galactose, lysophosphatidylcholine (LysoPC) (P-16:0), and tetradecenoyl-l-carnitine. The relative amounts of the four metabolites changed significantly and consistently after GEM monotherapy or combination therapy. The levels of these four metabolites were significantly different in the GEM-S and GEM-R pancreatic carcinoma xenograft models; thus, these metabolites could be effective predictive indicators of the efficacy of chemotherapy in PC patients, regardless of the administration of GEM alone or GEM plus nab-PTX.

## Introduction

Pancreatic cancer (PC) is one of the most aggressive human cancers, and affected patients have a 5 years survival rate of <9% (1, 2). Although tumor resection is the most curative option for PC patients, no more than 20% of patients can undergo surgery at the time of diagnosis because metastasis has already occurred (3). Therefore, chemotherapy plays a critical role in the therapeutic management of PC in patients with unresectable PC (4–6). Recent years have witnessed the rapid development of revolutionary targeted therapies and immune therapies, but these therapies have not shown significant results in PC (7–9). Thus, cytotoxic drugs remain the backbone of treatment for PC.

Gemcitabine (GEM) was approved as a the first-line drug for PC treatment in 1997, but the overall survival time of PC patients on GEM monotherapy is <6 months (10). Recently, GEM combinations were confirmed to increase median survival (11). GEM plus nanoparticle albumin-binding paclitaxel (nab-PTX) doublet therapy is a representative PC treatment that has been shown to increase overall survival at 1 and 2 years and to increase median survival by 1.8 months (12, 13). Thus, far, GEM monotherapy and combination therapy are the major therapeutic regimens for PC patients. However, chemotherapy resistance remains a key challenge in PC treatment (14, 15). In addition to cancer cells, the tumor microenvironment and pharmacokinetics contribute to clinical chemotherapy failure and apparent drug resistance (16–18).

Extracellular vesicles (EVs) have the potential to target specific tumor cells and affect tumor formation and progression; thus, EVs have been applied in the development of new therapeutic strategies to increase the efficacy of antitumor therapies (19). Further studies suggested that exosomes have the potential to stimulate an antitumor immune response (20, 21). Lucien et al. reported the application of EVs in tumor therapy (22). These tools can markedly improve the effectiveness of drug therapy. As a nanoparticle, nab-PTX is transported across the endothelial cell layer through EVs via biological albumin pathways and penetrates tumor tissue to increase the antitumor effect of PTX (23). However, potential predictive biomarkers of the effectiveness of chemotherapy in PC need to be explored to both better predict the chemotherapy response and avoid drug resistance.

Metabolomics is helpful for understanding systematic physiological responses induced by external stimuli. Thus, unbiased profiling can be used to identify metabolic features before and during treatment to identify outcomes related to treatment (24, 25) and to enhance the understanding and/or prediction of chemotherapeutic efficacy (26–29).

Considering the scientific rationale and the need to discover predictive biomarkers of the efficacy of GEM-based chemotherapy, we generated GEM-sensitive (GEM-S) and GEM-resistant (GEM-R) pancreatic carcinoma xenograft models in nude mice and analyzed the metabolic profiles in serum, liver, and tumor samples after treatment with GEM alone or GEM plus nab-PTX to identify potential metabolic biomarkers. Using pharmacometabolomics techniques, we explored potential predictive biomarkers of the efficacy of GEM-based therapy with the goal of achieving the optimal therapeutic results in PC patients.

## Materials and Methods

### Chemicals and Reagents

Fetal bovine serum was purchased from Invitrogen (Carlsbad, USA), and RPMI 1640 and DMEM media were supplied by Gibco (Thermo Fisher Scientific, China). GEM (Hubei Halfsky Pharmaceuticals Co., China) and albumin-binding PTX (Abraxane®, Abraxis BioScience, LLC, USA) were acquired from the pharmacy at Harbin Medical University Cancer Hospital. Deionized water was produced by a Milli-Q ultrapure water system (Millipore, Billerica, USA).

High-performance liquid chromatography-grade acetonitrile was purchased from Fisher Scientific (Waltham, MA, USA), and formic acid and high-performance liquid chromatography-grade methanol were purchased from Sigma-Aldrich and Fluka (St. Louis, MO, USA).

### Cell Culture

The human BxPC-3 and PANC-1 PC cell lines were purchased from Shanghai Institutes for Biological Sciences (Shanghai, China). BxPC-3 cells were cultured in RPMI-1640 medium, and PANC-1 cells were grown in DMEM. Both media were supplemented with 10% fetal bovine serum. These human PC cells were incubated at 37°C in 5% CO_2_. BxPC-3 cells are relatively responsive to GEM, whereas PANC-1 cells are relatively resistant to GEM (30–32). The cells were used in experiments in the logarithmic growth phase and were conformed to be pathogen free.

### Subcutaneous Tumor Growth Study

Male BALB/c nude mice (3–5 weeks old) were purchased from Vital River Laboratories (Beijing, China) to establish the subcutaneous xenograft model. The mice were housed in standard mouse plexiglass cages at 25 ± 1°C with 40–60% humidity and a 12/12-h light/dark cycle and were fed *ad-libitum* a regular autoclaved chow diet and water. Animal experiments were performed in accordance with the Ethics of Animal Experiments Committee of Harbin Institutes of Technology (Harbin, China).

BxPC-3 and PANC-1 cells were cultured and injected subcutaneously into BALB/c nude mice (right lower back) at a dose of 5 × 10^6^ cells per mouse in 0.1 ml of a 1:1 PBS/Matrigel mixture. When all mice had measurable tumors with an average volume of 100–150 mm^3^ (calculated as (length × width^2^)/2), BxPC-3 and PANC-1 tumor-bearing nude mice were randomly divided into three groups (*n* ≥ 6), and the treatment regimen was started immediately. The three groups of tumor-bearing mice were treated with GEM alone (G group), GEM plus nab-PTX (GP group), or normal saline (C group). The mice in the G group were treated with 30 mg/kg GEM twice a week (the days 1 and 4) for 2 weeks by intraperitoneal (i.p.) perfusion. The mice in the GP group were treated with GEM as stated for the G group and with 20 mg/kg nab-PTX once a week (day 4) for 2 weeks by intravenous (i.v.) injection through the tail vein. The mice in the C group were injected with 100 μl of normal saline solution through the same route and on the same days as in the G group. After initiating treatment, the tumors were measured every other day until the mice were killed. The relative tumor volume was calculated by dividing the tumor volume at each time point by the tumor volume at the start of treatment. Net tumor growth was calculated by subtracting the tumor volume on the first treatment day from that on the last day. BxPC-3 and PANC-1 tumor-bearing nude mice treated with GEM monotherapy or combination therapy were killed after 2 weeks of treatment. Whole blood, liver, and tumor samples were collected and processed for metabolite analysis.

### Sample Collection and Preparation

Whole blood samples were collected from tumor-bearing mice in non-anticoagulant vacuum tubes. Then, the samples were centrifuged at 4,000 × *g* to separate the serum for metabolic profiling. A volume of 300 μl of precooled methanol/acetonitrile (1:1) was added to 100 μl of serum to precipitate the protein. The samples were then placed in a rotary vacuum to obtain a dry residue, which was stored at −80°C until analysis.

The tumor and liver samples were resected from tumor-bearing mice, washed immediately with cold saline, and dried on filter paper. Then, the tissues were stored in liquid nitrogen until further processing. Before the metabolite analysis, 20 mg of frozen tissue was sliced at a thickness of 20 μm and incubated in 75% precooled methanol solution at a concentration of 20 μl/mg. The tissue sections were subjected to ultrasonic disruption in 5-s pulses alternating with 5-s pauses, for a total of 2 min. The tissues were then centrifuged at 12,000 rpm for 15 min at 4°C. The supernatant was evaporated by rotary vacuum and stored at −80°C until further analysis. This study was approved by the Ethics of Animal Experiments Committee of Harbin Institutes of Technology.

### UPLC-Q/TOF for Non-targeted Metabolomic MS Analysis

The samples were redissolved in 100 μl of 50% methanol, and 5 μl of the supernatant was injected into a BEH C_18_ column (2.1 mm × 100 mm, 1.7 μm; Waters, Milford, USA) on an ultraperformance liquid chromatography (UPLC) system (Waters, Milford, USA) with a flowrate of 0.35 ml/min and a column temperature of 40°C. The mobile phase conditions were as follows: linear gradient analysis with mobile phase A, acetonitrile containing 0.1% formic acid, and mobile phase B, 0.1% formic acid. The ratio of eluting solvent A was maintained at 1% for 0.5 min and then linearly increased from 1 to 53% from 0.5 to 3.5 min, to 70% from 3.5 to 7.5 min, and to 90% from 7.5 to 9 min. Then, 90% eluting solvent A was maintained for 4 min. Finally, the ratio of eluting solvent A was linearly decreased from 90 to 1% from 13 to 15 min.

Mass spectrum (MS) acquisition and MS/MS identification were both performed in positive and negative modes with a 6520 series accurate quadrupole time-of-flight mass spectrometer (Q-TOF MS) equipped with a dual electrospray ion source (Agilent, Santa Clara, CA, USA). For MS, the instrument was operated using an electrospray ionization source in both positive and negative ionization modes with survey scans acquired from *m*/*z* 70 to 1,100 at a scan rate of 1.5 spectra/s. The ionization parameters were as follows: capillary voltage, 4.5 kV in positive mode and 3.5 kV in negative mode; gas temperature, 330°C; gas flowrate; 10 L/min; fragment voltage, 100 V; and skimmer voltage, 65 V.

### Metabolite Identification

Differential metabolite structural information, including the retention time (RT), *m/z* and MS/MS spectra, was confirmed using spectra from the Human Metabolome Database (http://www.hmdb.ca/), Metabolite and Tandem MS Database (http://metlin.scripps.edu/index.php), or Mass Bank Database (http://www.massbank.jp/).

### Statistical Analysis

Both principal component analysis (PCA) and partial least squares discriminant analysis (PLS-DA) were performed to obtain the global metabolic profiles in serum, liver, and tumor samples from BxPC-3 or PANC-1 tumor-bearing nude mice, in the G, GP, and C groups. To avoid overfitting, we used cross-validation to certify the stability and credibility of the PLS-DA models. Furthermore, hierarchical cluster analysis (HCA) was performed to visualize the changes in these metabolites after GEM monotherapy or combination therapy in BxPC-3 or PANC-1 PC tumor-bearing nude mice.

The relative amounts of the metabolites with discriminatory significance were calculated by integrating their characteristic signals in the MS spectra. One-way ANOVA was employed to determine the significance of differences in each metabolite among treatment groups in both BxPC-3 and PANC-1 tumor-bearing nude mice. Multivariate statistical analysis was performed using SIMCA-p v.11.5 (Umetrics AB, Umea, Sweden) for PCA, PLS-DA, and cross-validation. HCA was conducted by the R package *tnet* for standardization and hierarchical clustering (33).

## Results

### Establishing GEM-S and GEM-R Subcutaneous PC Xenograft Models

**Figure 1:**
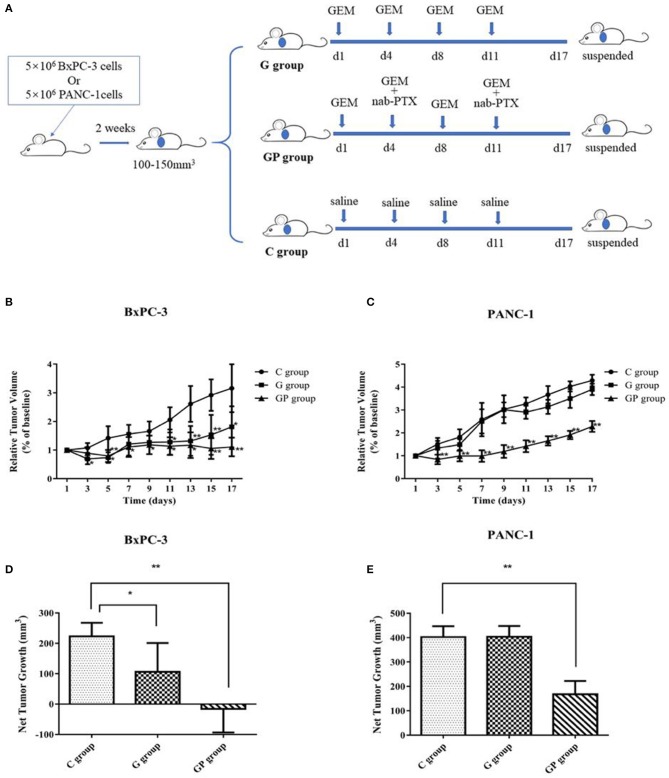
The impact of gemcitabine (GEM)-based chemotherapies on GEM-sensitive and GEM-resistant pancreatic subcutaneous tumor xenografts. **(A)** The scheme of the treatment performed *in vivo*. **(B)** Relative tumor volume changes of BxPC-3 tumor-bearing mice with GEM-based chemotherapy. **(C)** Relative tumor volume changes of PANC-1 tumor-bearing mice with GEM-based chemotherapy. **(D)** Net tumor growth changes of BxPC-3 tumor-bearing mice with GEM-based chemotherapy. **(E)** Net tumor growth changes of PANC-1 tumor-bearing mice with GEM-based chemotherapy. Data are representative of mean values ± standard deviation per group. **Significant difference (*P* < 0.01) compared with C group, *significant difference (*P* < 0.05) compared with C group. G, GEM; GP, GEM plus nab-PTX; C, untreated.

In this study, GEM-S and GEM-R subcutaneous tumor xenograft models were generated by the subcutaneous injection human BxPC-3 or PANC-1 PC cells into BALB/c nude mice. After 2 weeks, both GEM-S and GEM-R tumor xenografts were established, and the tumor-bearing nude mice were treated with GEM alone or GEM plus nab-PTX for 2 weeks. The treatment scheme is shown in Figure 1A. The tumor-bearing mice remained in good condition during the treatment. All the mice treated with GEM alone or GEM plus nab-PTX survived until the end of the 17 days observation period. The mice weighed between 15 and 20 g throughout the treatment period, with no statistically significant differences in body weight among treatment groups. The changes in the subcutaneous tumor xenografts were investigated in mice with different drug-resistant phenotypes after GEM monotherapy or combination therapy. In GEM-S tumor-bearing nude mice, both GEM monotherapy and GEM plus nab-PTX doublet therapy significantly inhibited tumor growth, as determined by relative and net tumor growth (Figures 1B,D). The net tumor growths in the G and GP groups were 106.09 mm^3^ (*P* < 0.05) and −15.72 mm^3^ (*P* < 0.001), respectively. Furthermore, GEM combination therapy had stronger inhibitory activity than GEM monotherapy. However, in GEM-R tumor-bearing nude mice, tumor growth was not significantly affected by GEM monotherapy compared with saline but was significantly suppressed by GEM plus nab-PTX. The net tumor growths in the GP and G groups were 167.82 mm^3^ (*P* < 0.001) and 403.61 mm^3^ (*P* > 0.05), respectively (Figures 1C,E). These results indicated different efficiencies with GEM monotherapy in the GEM-S and GEM-R subgroups, but the similar efficiencies of GEM combination therapy in the two subgroups, suggesting the successful establishment of GEM-S and GEM-R tumor models.

### Metabolic Profiling of GEM-S and GEM-R Tumor-Bearing Nude Mice Treated With GEM-Based Chemotherapy

Metabolites were identified by comparing RT, *m/z* values, and MS fragmentation patterns with published data. In total, this study identified 88 and 79 metabolites in serum, 78 and 87 metabolites in liver, 83 and 94 metabolites in the tumors from GEM-S (BxPC-3) and GEM-R (PANC-1) tumor-bearing mice, respectively.

**Figure 2:**
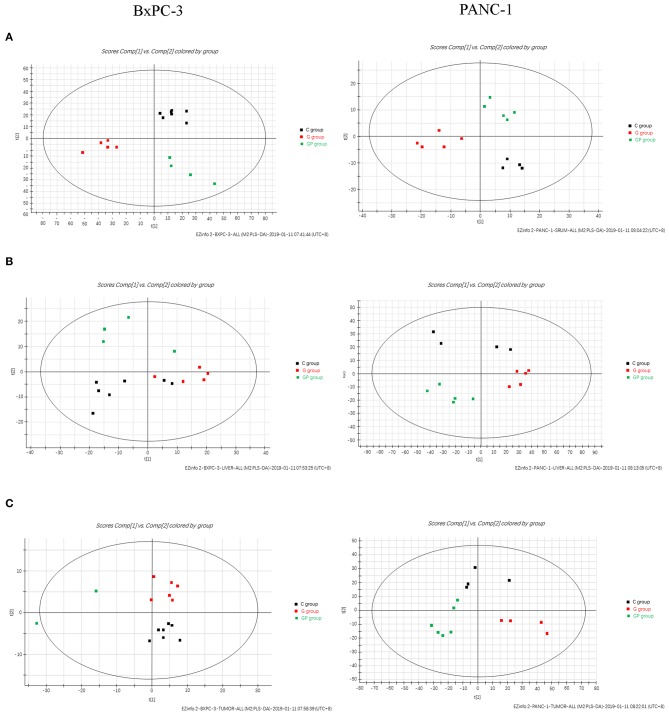
The two-dimensional score plots of PLS-DA of serum, liver, and tumor samples to discriminate among GEM plus nab-PTX, GEM alone, and untreated therapy in BxPC-3 and PANC-1 pancreatic tumor-bearing mice. **(A)** Serum samples; **(B)** liver samples; **(C)** tumor samples.

To provide comparative interpretations and to visualize metabolic similarities or differences among GEM monotherapy, GEM combination therapy, and control therapy in GEM-S and GEM-R tumor-bearing mice, the UPLC/Q-TOF MS spectra datasets of serum, liver, and tumor were separately analyzed by multivariate analysis. Both unsupervised PCA and supervised PLS-DA multivariate analyses were conducted to provide an overview of all samples in a data set. The PCA score plots for different tissues in GEM-S/R tumor-bearing mice treated with different regimens had no obvious outlier (Supplemental Figure 1). The PLS-DA score plot clearly showed the formation of independent clusters among the GEM monotherapy, GEM plus nab-PTX doublet therapy, and control groups of serum, liver, and tumor samples from GEM-S and GEM-R tumor-bearing mice (Figure 2). The model statistics, *R*^2^ and *Q*^2^, indicated that the models were robust without statistical overfitting (Supplemental Figure 2). These data suggested that the metabolic profiles in the serum, livers, and tumors of GEM-S and GEM-R pancreatic tumor-bearing mice were clearly different in each treatment group, indicating significant differences in the overall metabolism in the serum, livers, and tumors of tumor-bearing mice with different GEM-resistant phenotypes upon exposure to GEM monotherapy or combination therapy.

**Figure 3:**
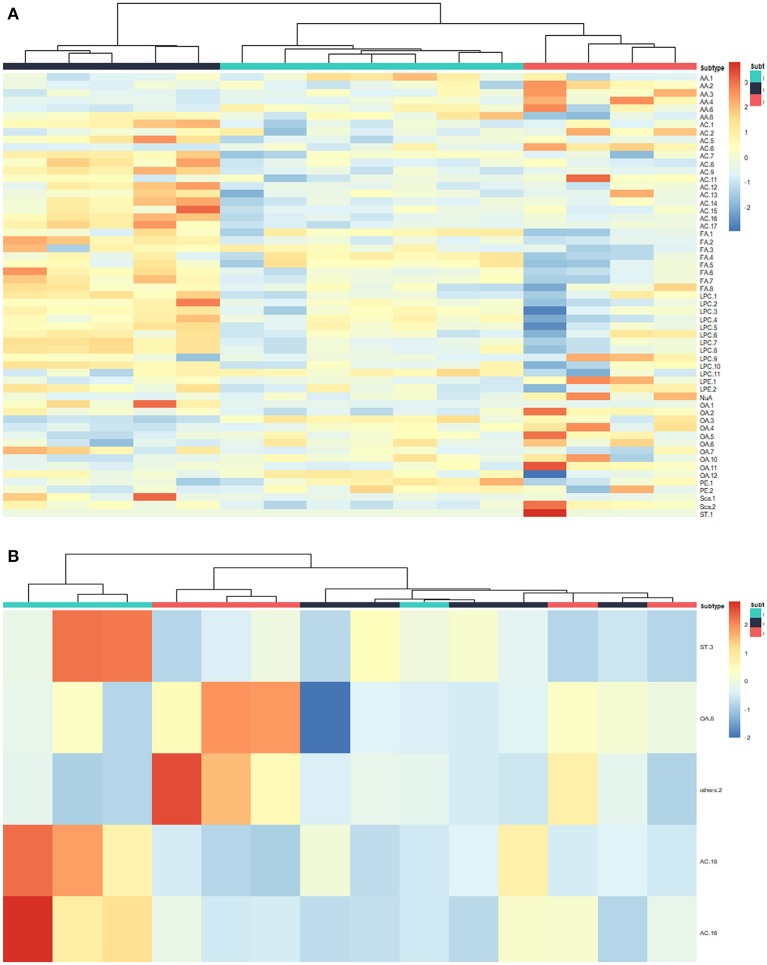
Hierarchical cluster analysis (HCA) heatmap of the differential metabolites in serum samples treated by GEM plus nab-PTX or GEM in GEM-S subgroup **(A)** and GEM-R subgroup **(B)**.

To further explore the significance of different metabolites in serum, liver, and tumor samples from different treatment groups of GEM-S and GEM-R tumor-bearing mice, one-way ANOVA was employed. HCA as performed to visualize the changes in differential metabolites in different tissues from GEM-S/R PC tumor-bearing nude mice treated with GEM monotherapy or combination therapy. As shown in the serum HCA heatmap, the observations in the GEM-S treatment subgroups were completely separated, but the separation in the GEM-R subgroups was not obvious. The HCA heatmap for all differential metabolites in serum samples from the GEM-S and GEM-R treatment subgroups is presented in Figure 3, and the differentially expressed metabolites are summarized in Table 1. The differential tumor and liver metabolites in the GEM-S and GEM-R subgroups treated with GEM-based chemotherapy were not significantly separated in the HCA-heatmap, as shown in Supplemental Figures 3, 4. The significant differential metabolites in liver and tumor samples from mice treated with GEM alone or GEM plus nab-PTX are listed in Supplemental Tables 1–4. The results described above suggested that among the three types of samples, the serum metabolites showed the most significant differences between GEM-S and GEM-R subgroups treated with GEM monotherapy or combination therapy. Therefore, metabolic changes in serum could successfully reflect the efficacy of GEM-based treatment.

**Table 1:** Summary of the differential metabolites in GEM-S/R pancreatic tumor-bearing mice treated with GEM-based chemotherapy.

**Name**	**Group**	**Molecular** **formula**	**Ion (*m*/*z*)**	**RT/min**	**ESI mode**
l-Arginine	AA-1	C_6_H_14_N4O_2_	173.1022	1.55	M – H
l-Glutamine	AA-2	C_5_H_10_N_2_O_3_	145.0612	1.60	M – H
l-Kynurenine	AA-3	C_10_H_12_N_2_O_3_	207.0760	2.92	M – H
l-Methionine	AA-4	C_5_H_11_NO_2_S	150.0582	2.21	M + H
l-Phenylalanine	AA-5	C_9_H_11_NO_2_	164.0715	3.45	M – H
l-Threonine	AA-6	C_4_H_9_NO_3_	118.0503	1.58	M – H
L-Tryptophan	AA-7	C_11_H_12_N_2_O_2_	205.0972	3.69	M + H
l-Tyrosine	AA-8	C_9_H_11_NO_3_	182.0809	2.82	M + H
Acetyl-l-carnitine	AC-1	C_9_H_17_NO_4_	204.1228	1.66	M + H
Butyryl-l-carnitine	AC-2	C_11_H_21_NO_4_	232.1539	3.27	M + H
Decanoyl-l-carnitine	AC-3	C_17_H_33_NO_4_	316.2473	5.58	M + H
Decenoyl-l-carnitine	AC-4	C_17_H_31_NO_4_	314.2314	5.46	M + H
Dodecanoyl-l-carnitine	AC-5	C_19_H_37_NO_4_	344.2788	5.68	M + H
Glutaryl-l-carnitine	AC-6	C_12_H_21_NO_6_	276.1417	2.91	M + H
Hexadecanoyl-l-carnitine	AC-7	C_23_H_45_NO_4_	400.3449	7.79	M + H
Hexadecenoyl-l-carnitine	AC-8	C_23_H_43_NO_4_	398.3264	7.02	M + H
Hexanoyl-l-carnitine	AC-9	C_13_H_25_NO_4_	260.1845	4.00	M + H
Hexenoyl-l-carnitine	AC-10	C_13_H_23_NO_4_	258.1699	5.29	M + H
l-Carnitine	AC-11	C_7_H_15_NO_3_	162.1125	1.60	M + H
Octadecadienyl-l-carnitine	AC-12	C_25_H_45_NO_4_	424.3411	7.40	M + H
Octadecanoyl-l-carnitine	AC-13	C_25_H_49_NO_4_	428.3724	9.17	M + H
Octadecenoyl-l-carnitine	AC-14	C_25_H_47_NO_4_	426.3569	8.21	M + H
Tetradecadienyl-l-carnitine	AC-15	C_21_H_37_NO_4_	368.2785	5.64	M + H
Tetradecanoyl-l-carnitine	AC-16	C_21_H_41_NO_4_	372.3096	6.62	M + H
Tetradecenoyl-l-carnitine	AC-17	C_21_H_39_NO_4_	370.2954	6.09	M + H
Valeryl-l-carnitine	AC-18	C_12_H_23_NO_4_	246.1699	3.58	M + H
(Iso)leucyl-phenylalanine	DP-1	C_15_H_22_N_2_O_3_	279.1746	2.11	M + H
Glycyl-phenylalanine	DP-2	C_11_H_14_N_2_O_3_	221.0916	3.59	M – H
Histidinyl-cysteine	DP-3	C_9_H_14_N_4_O_3_S	259.0889	1.93	M + H
(±)4-HDoHE	FA-1	C_22_H_32_O_3_	343.2267	8.22	M – H
(±)5-HETE	FA-2	C_20_H_32_O_3_	319.2220	11.62	M – H
(R)-3-Hydroxy-hexadecanoic acid	FA-3	C_16_H_32_O_3_	271.2264	8.19	M – H
20-OH-LTB4	FA-4	C_20_H_32_O_4_	335.2218	7.28	M – H
9-Octadecenoic acid	FA-5	C_18_H_33_FO_2_	345.2425	9.10	M + FA – H
Arachidonic acid	FA-6	C_20_H_32_O_2_	303.2322	10.76	M – H
Docosahexaenoic acid	FA-7	C_22_H_32_O_2_	327.2323	10.60	M – H
EPA	FA-8	C_20_H_30_O_2_	301.2163	10.22	M – H
1-Linoleoyl glycerophosphocholine	LPC-1	C_26_H_50_NO_7_P	520.3404	7.61	M + H
LysoPC(14:0)	LPC-2	C_22_H_46_NO_7_P	468.3106	9.53	M + H
LysoPC(16:0)	LPC-3	C_24_H_50_NO_7_P	496.3402	8.46	M + H
LysoPC(17:0)	LPC-4	C_25_H_52_NO_7_P	554.3449	9.23	M + FA – H
LysoPC(18:0)	LPC-5	C_26_H_54_NO_7_P	524.3716	10.17	M + H
LysoPC(18:2)	LPC-6	C_26_H_50_NO_7_P	564.3309	7.62	M + FA – H
LysoPC(20:3)	LPC-7	C_28_H_52_NO_7_P	568.3404	7.86	M + Na
LysoPC(20:4)	LPC-8	C_28_H_50_NO_7_P	588.3302	7.61	M + FA – H
LysoPC(20:5)	LPC-9	C_28_H_48_NO_7_P	542.3233	7.13	M + H
LysoPC(22:6)	LPC-10	C_30_H_50_NO_7_P	612.3300	7.52	M + FA – H
LysoPC(P-16:0)	LPC-11	C_24_H_50_NO_6_P	524.3342	8.79	M + FA – H
PC(O-16:0/0:0)	LPC-12	C_23_H_48_NO_7_P	482.3235	10.87	M + H
LysoPE(0:0/20:5)	LPE-1	C_25_H_42_NO_7_P	500.2783	7.06	M + H
LysoPE(20:2)	LPE-2	C_25_H_48_NO_7_P	504.3077	7.62	M – H
Succinoadenosine	NuA	C_14_H_17_N_5_O_8_	382.0974	2.94	M – H
Uridine	NuC	C_9_H_12_N_2_O_6_	245.0732	1.93	M + H
2-Methyl-3-hydroxypropanoate	OA-1	C_4_H_8_O_3_	103.0401	2.69	M – H
3-Hydroxyadipic acid	OA-2	C_6_H_10_O_5_	161.0442	1.57	M – H
3-Indolelactic acid	OA-3	C_10_H_9_NO	204.0659	4.03	M + FA – H
Citric acid	OA-4	C_6_H_8_O_7_	191.0193	2.17	M – H
Fumaric acid	OA-5	C_4_H_4_O_4_	115.0035	2.30	M – H
Gluconic acid	OA-6	C_6_H_12_O_7_	195.0498	1.64	M – H
l-2-Aminoadipic acid	OA-7	C_6_H_11_NO_4_	162.0760	2.84	M + H
Lactic acid	OA-8	C_3_H_6_O_3_	91.0396	2.17	M + H
l-Glutamate	OA-9	C_5_H_9_NO_4_	146.0462	2.05	M – H
Malic acid	OA-10	C_4_H_6_O_5_	133.0142	1.89	M – H
Pyrroline hydroxycarboxylic acid	OA-11	C_5_H_7_NO_3_	130.0505	1.57	M + H
Uric acid	OA-12	C_5_H_4_N_4_O_3_	169.0353	2.39	M + H
(10E)-9-Oxo-10-hexadecenoic acid	Others-1	C_16_H_28_O_3_	269.2111	8.01	M + H
Succinic anhydride	Others-2	C_4_H_4_O_3_	101.0239	2.80	M + H
PE(20:0/20:4)	PE-1	C_45_H_82_NO_8_P	840.5732	8.66	M + FA – H
PE(P-18:0/0:0)	PE-2	C_23_H_48_NO_6_P	464.3131	10.35	M – H
1-[(5-Amino-5-carboxypentyl)amino]−1-deoxyfructose	Sca-1	C_12_H_24_N_2_O_7_	353.1441	10.77	M + FA – H
D-galactose	Sca-2	C_6_H_12_O_6_	179.0552	1.57	M – H
3α,7α-Dihydroxy-5beta-cholestan-26-al	ST-1	C_26_H_42_O_4_	463.3055	5.34	M + FA – H
7α-Hydroxycholest-4-en-3-one	ST-2	C_27_H_44_O_2_	445.3314	11.80	M + FA – H
Chenodeoxycholate	ST-3	C_24_H_40_O_4_	391.2832	6.80	M – H
Glycocholate	ST-4	C_26_H_43_NO_6_	466.3166	10.96	M + H

*RT, retention time; ESI, electrospray ionization*.

### Analysis of Serum Metabolites Correlated With GEM-Based Chemotherapy Efficacy

To investigate the relationship between the efficacy of GEM-based chemotherapy and changes in serum metabolites, we analyzed the differential metabolites in serum samples in the GEM-S and GEM-R subgroups when GEM administered alone or in combination. In the GEM monotherapy group, 39 differential metabolites were identified in the GEM-S subgroup, but only one differential metabolite (tetradecanoyl-l-carnitine) was found in the GEM-R subgroup, this metabolite was also identified in the GEM-S subgroup. Thus, there were 38 differential metabolites in the GEM-R and GEM-S subgroups in response to GEM alone, as shown in Table 2.

**Table 2:** Details of 38 significant differential metabolites in serum treated with GEM alone.

**Name**	**Group**	**Molecular** **formula**	**Ion** **(*m*/*z*)**	**RT/** **min**	**ESI** **mode**	**Cells**	**Relative amount**		**Tendency** **G/C**
							**C group**	**G group**	
l-Arginine	AA-1	C_6_H_14_N_4_O_2_	173.1022	1.55	M – H	BxCP-3	9875.00 ± 3836.49	5, 386.98 ± 1744.77	↓^*^
l-Threonine	AA-6	C_4_H_9_NO_3_	118.0503	1.58	M – H	BxCP-3	26532.48 ± 4627.81	15, 844.89 ± 1636.40	↓^**^
Acetyl-l-carnitine	AC-1	C_9_H_17_NO_4_	204.1228	1.66	M + H	BxCP-3	968263.26 ± 187007.55	1, 740, 917.03 ± 269976.93	↑^**^
Dodecanoyl-l-carnitine	AC-5	C_19_H_37_NO_4_	344.2788	5.68	M + H	BxCP-3	11339.49 ± 3530.29	25, 546.56 ± 6110.40	↑^**^
Hexadecanoyl-l-carnitine	AC-7	C_23_H_45_NO_4_	400.3449	7.79	M + H	BxCP-3	119228.98 ± 58879.02	220, 688.89 ± 30650.67	↑^**^
Hexadecenoyl-l-carnitine	AC-8	C_23_H_43_NO_4_	398.3264	7.02	M + H	BxCP-3	10802.06 ± 5054.19	32, 057.59 ± 10376.39	↑^**^
Hexanoyl-l-carnitine	AC-9	C_13_H_25_NO_4_	260.1845	4.00	M + H	BxCP-3	16851.57 ± 7732.15	57, 698.54 ± 10272.46	↑^**^
Octadecadienyl-l-carnitine	AC-12	C_25_H_45_NO_4_	424.3411	7.40	M + H	BxCP-3	45110.71 ± 11369.72	107, 940.11 ± 38077.38	↑^**^
Octadecanoyl-l-carnitine	AC-13	C_25_H_49_NO_4_	428.3724	9.17	M + H	BxCP-3	55950.37 ± 12841.99	75, 960.78 ± 12611.71	↑^*^
Octadecenoyl-l-carnitine	AC-14	C_25_H_47_NO_4_	426.3569	8.21	M + H	BxCP-3	149743.14 ± 44891.11	371, 505.85 ± 98606.22	↑^**^
Tetradecadienyl-l-carnitine	AC-15	C_21_H_37_NO_4_	368.2785	5.64	M + H	BxCP-3	10972.41 ± 2482.95	20, 093.36 ± 7141.80	↑^*^
Tetradecenoyl-l-carnitine	AC-17	C_21_H_39_NO_4_	370.2954	6.09	M + H	BxCP-3	28793.33 ± 6584.81	74, 972.52 ± 17484.96	↑^**^
(±)5-HETE	FA-2	C_20_H_32_O_3_	319.2220	11.62	M – H	BxCP-3	8253.37 ± 3628.19	27, 734.61 ± 6535.84	↑^**^
Arachidonic Acid	FA-6	C_20_H_32_O_2_	303.2322	10.76	M – H	BxCP-3	760719.54 ± 177843.33	1, 423, 954.30 ± 339959.20	↑^**^
Docosahexaenoic acid	FA-7	C_22_H_32_O_2_	327.2323	10.60	M – H	BxCP-3	735512.08 ± 160556.41	1, 244, 330.77 ± 239534.02	↑^**^
EPA	FA-8	C_20_H_30_O_2_	301.2163	10.22	M – H	BxCP-3	19434.48 ± 3129.90	25, 432.95 ± 2747.76	↑^*^
1-Linoleoyl glycerophosphocholine	LPC-1	C_26_H_50_NO_7_P	520.3404	7.61	M + H	BxCP-3	2896473.15 ± 505504.68	5, 048, 468.70 ± 543989.97	↑^**^
LysoPC(16:0)	LPC-3	C_24_H_50_NO_7_P	496.3402	8.46	M + H	BxCP-3	42578974.33 ± 6563740.46	50, 915, 486.29 ± 2657063.51	↑^*^
LysoPC(18:0)	LPC-5	C_26_H_54_NO_7_P	524.3716	10.17	M + H	BxCP-3	36522390.14 ± 5936429.38	45, 787, 178.78 ± 4885002.63	↑^*^
LysoPC(18:2)	LPC-6	C_26_H_50_NO_7_P	564.3309	7.62	M + FA – H	BxCP-3	6850986.07 ± 770086.97	8, 701, 798.30 ± 756555.84	↑^**^
LysoPC(20:3)	LPC-7	C_28_H_52_NO_7_P	568.3404	7.86	M + Na	BxCP-3	2543660.08 ± 502776.55	4, 978, 523.14 ± 408274.04	↑^**^
LysoPC(20:4)	LPC-8	C_28_H_50_NO_7_P	588.3302	7.61	M + FA – H	BxCP-3	2123610.42 ± 512749.34	3, 681, 731.23 ± 261319.03	↑^**^
LysoPC(22:6)	LPC-10	C_30_H_50_NO_7_P	612.3300	7.52	M + FA – H	BxCP-3	1229698.44 ± 258896.99	1, 796, 716.66 ± 119318.24	↑^**^
LysoPC(P-16:0)	LPC-11	C_24_H_50_NO_6_P	524.3342	8.79	M + FA – H	BxCP-3	34629.60 ± 2605.61	28, 986.96 ± 5201.97	↓^*^
LysoPE(20:2)	LPE-2	C_25_H_48_NO_7_P	504.3077	7.62	M – H	BxCP-3	55589.38 ± 7834.65	69, 930.71 ± 7487.69	↑^*^
2-Methyl-3-hydroxypropanoate	OA-1	C_4_H_8_O_3_	103.0401	2.69	M – H	BxCP-3	55227.31 ± 5328.46	147, 658.95 ± 60997.03	↑^**^
3-Hydroxyadipic acid	OA-2	C_6_H_10_O_5_	161.0442	1.57	M – H	BxCP-3	54113.53 ± 7009.07	68, 401.75 ± 6679.22	↑^**^
3-Indolelactic acid	OA-3	C_10_H_9_NO	204.0659	4.03	M + FA – H	BxCP-3	57440.71 ± 7174.73	32, 644.46 ± 2279.59	↓^**^
Citric acid	OA-4	C_6_H_8_O_7_	191.0193	2.17	M – H	BxCP-3	496393.03 ± 78211.54	360, 236.87 ± 44489.98	↓^**^
Fumaric acid	OA-5	C_4_H_4_O_4_	115.0035	2.30	M – H	BxCP-3	32057.27 ± 8599.32	15, 938.37 ± 7620.63	↓^*^
Gluconic acid	OA-6	C_6_H_12_O_7_	195.0498	1.64	M – H	BxCP-3	68632.55 ± 21169.23	39, 013.18 ± 17157.22	↓^*^
l-2-Aminoadipic acid	OA-7	C_6_H_11_NO_4_	162.0760	2.84	M + H	BxCP-3	29088.93 ± 9664.36	63, 599.34 ± 27102.05	↑^*^
Malic acid	OA-10	C_4_H_6_O_5_	133.0142	1.89	M – H	BxCP-3	20401.69 ± 5428.88	10, 278.65 ± 3096.83	↓^**^
PE(20:0/20:4)	PE-1	C_45_H_82_NO_8_P	840.5732	8.66	M + FA – H	BxCP-3	90777.79 ± 37667.35	44, 074.25 ± 21664.45	↓^*^
PE(P-18:0/0:0)	PE-2	C_23_H_48_NO_6_P	464.3131	10.35	M – H	BxCP-3	97718.87 ± 15391.83	77, 845.82 ± 10044.57	↓^*^
1-[(5-Amino-5-carboxypentyl)amino]-1-deoxyfructose	Sca-1	C_12_H_24_N_2_O_7_	353.1441	10.77	M + FA – H	BxCP-3	15577.42 ± 8189.33	105, 471.51 ± 91089.15	↑^*^
D-galactose	Sca-2	C_6_H_12_O_6_	179.0552	1.57	M – H	BxCP-3	480657.67 ± 52785.37	623, 507.87 ± 48777.64	↑^**^
3α,7α-Dihydroxy-5beta-cholestan-26-al	ST-1	C_26_H_42_O_4_	463.3055	5.34	M + FA – H	BxCP-3	1, 162.49 ± 538.49	105.76 ± 139.57	↓^**^

*Details of the 38 significant differential metabolites in serum after treatment with GEM alone, compared with no treatment. Relative amount in LC-MS were represented as mean values ± standard deviation*.

* *Significant difference (P < 0.05) compared with C group*.

** *Significant difference (P < 0.01) compared with C group*.

*RT, retention time; ESI, electrospray ionization; G, GEM; C, untreated*.

The impact of GEM plus nab-PTX doublet therapy on the differential metabolites in the GEM-S/R subgroups was also explored. A total of 21 differential metabolites were identified in the GEM-S subgroup, but only five were found in the GEM-R subgroup. None of the differential metabolites were shared between the two subgroups, so there were 26 different substances associated with GEM combination therapy, as shown in Table 3.

**Table 3:** Details of 26 significant differential metabolites in serum treated with GEM plus nab-PTX.

**Name**	**Group**	**Molecular formula**	**Ion (m/z)**	**RT/** **min**	**ESI mode**	**Cells**	**Relative amount**		**Tendency GP/C**
							**C group**	**GP group**	
l-Glutamine	AA-2	C_5_H_10_N_2_O_3_	145.0612	1.60	M – H	BxCP-3	100, 858.01 ± 26, 654.03	165, 680.92 ± 33, 971.42	↑^*^
l-Kynurenine	AA-3	C_10_H_12_N_2_O_3_	207.0760	2.92	M – H	BxCP-3	10, 289.70 ± 2, 792.33	18, 523.54 ± 6, 410.56	↑^*^
l-Methionine	AA-4	C_5_H_11_NO_2_S	150.0582	2.21	M + H	BxCP-3	1, 527, 308.62 ± 294, 582.65	2, 929, 715.33 ± 915, 061.74	↑^**^
l-Tyrosine	AA-8	C_9_H_11_NO_3_	182.0809	2.82	M – H	BxCP-3	1, 515, 704.13 ± 328, 839.50	970, 572.20 ± 224, 505.36	↓^*^
Butyryl-l-carnitine	AC-2	C_11_H_21_NO_4_	232.1539	3.27	M + H	BxCP-3	328, 765.96 ± 111, 974.77	531, 315.52 ± 154, 237.69	↑^*^
Glutaryl-l-carnitine	AC-6	C_12_H_21_NO_6_	276.1417	2.91	M + H	BxCP-3	1, 590.03 ± 1, 239.03	5, 617.75 ± 1, 394.57	↑^**^
l-Carnitine	AC-11	C_7_H_15_NO_3_	162.1125	1.60	M + H	BxCP-3	478, 734.52 ± 75, 644.77	666, 970.91 ± 163, 211.37	↑^*^
Tetradecanoyl-l-carnitine	AC-16	C_21_H_41_NO_4_	372.3096	6.62	M + H	PANC-1	48, 142.37 ± 24, 332.88	21, 425.17 ± 5, 220.49	↓^*^
Tetradecenoyl-l-carnitine	AC-17	C_21_H_39_NO_4_	370.2954	6.09	M + H	BxCP-3	28, 793.33 ± 6, 584.81	40, 284.70 ± 1, 940.42	↑^*^
Valeryl-l-carnitine	AC-18	C_12_H_23_NO_4_	246.1699	3.58	M + H	PANC-1	80, 376.63 ± 29, 982.71	33, 188.45 ± 5, 045.77	↓^**^
(±)4-HDoHE	FA-1	C_22_H_32_O_3_	343.2267	8.22	M – H	BxCP-3	153, 912.65 ± 56, 049.42	52, 282.64 ± 38, 203.78	↓^*^
(R)-3-Hydroxy-hexadecanoic acid	FA-3	C_16_H_32_O_3_	271.2264	8.19	M – H	BxCP-3	7, 816.33 ± 1, 749.10	4, 582.38 ± 1, 124.62	↓^*^
20-OH-LTB4	FA-4	C_20_H_32_O_4_	335.2218	7.28	M – H	BxCP-3	83, 417.60 ± 27, 509.39	23, 527.91 ± 18, 339.80	↓^**^
9-Octadecenoic acid	FA-5	C_18_H_33_FO_2_	345.2425	9.10	M + FA – H	BxCP-3	68, 422.44 ± 26, 889.19	26, 507.56 ± 21, 073.88	↓^*^
LysoPC(17:0)	LPC-4	C_25_H_52_NO_7_P	554.3449	9.23	M + FA – H	BxCP-3	395, 547.41 ± 64, 972.63	196, 621.61 ± 86, 953.89	↓^**^
LysoPC(20:5)	LPC-9	C_28_H_48_NO_7_P	542.3233	7.13	M + H	BxCP-3	56, 917.91 ± 8, 015.03	114, 121.17 ± 45, 269.36	↑^*^
LysoPC(P-16:0)	LPC-11	C_24_H_50_NO_6_P	524.3342	8.79	M + FA – H	BxCP-3	34, 629.60 ± 2, 605.61	23, 511.35 ± 4, 520.37	↓^**^
LysoPE(0:0/20:5)	LPE-1	C_25_H_42_NO_7_P	500.2783	7.06	M + H	BxCP-3	11, 551.74 ± 1, 758.15	23, 039.08 ± 7, 784.94	↑^**^
Succinoadenosine	NuA	C_14_H_17_N_5_O_8_	382.0974	2.94	M – H	BxCP-3	3, 640.45 ± 1, 127.16	8, 718.73 ± 3, 377.89	↑^**^
3-Hydroxyadipic acid	OA-2	C_6_H_10_O_5_	161.0442	1.57	M – H	BxCP-3	54, 113.53 ± 7, 009.07	85, 338.89 ± 17, 658.29	↑^**^
Lactic acid	OA-8	C_3_H_6_O_3_	91.0396	2.17	M + H	PANC-1	1, 222, 994.07 ± 281, 880.94	2, 032, 773.50 ± 563, 307.86	↑^*^
Pyrroline hydroxycarboxylic acid	OA-11	C_5_H_7_NO_3_	130.0505	1.57	M + H	BxCP-3	58, 256.40 ± 14, 372.76	106, 881.93 ± 37, 585.06	↑^*^
Uric acid	OA-12	C_5_H_4_N_4_O_3_	169.0353	2.39	M + H	BxCP-3	1, 643, 466.59 ± 278, 101.45	934, 122.96 ± 535, 837.31	↓^*^
d-Galactose	Sca-2	C_6_H_12_O_6_	179.0552	1.57	M – H	BxCP-3	480, 657.67 ± 52, 785.37	746, 257.70 ± 151, 525.57	↑^**^
Chenodeoxycholate	ST-3	C_24_H_40_O_4_	391.2832	6.80	M – H	PANC-1	26230.45 ± 15, 995.87	3, 139.13 ± 4, 144.19	↓^*^
Succinic anhydride	Others-2	C_4_H_4_O_3_	101.0239	2.80	M + H	PANC-1	16567.11 ± 5, 262.97	40971.71 ± 19040.09	↑^*^

*Details of the 26 significant differential metabolites in serum after treatment with GEM plus nab-PTX, compared with no treatment. Relative amount in LC-MS were represented as mean values ± standard deviation*.

* *Significant difference (P < 0.05) compared with C group*.

** *Significant difference (P < 0.01) compared with C group*.

*RT, retention time; ESI, electrospray ionization; GP, GEM plus nab-PTX; C, untreated*.

**Figure 4:**
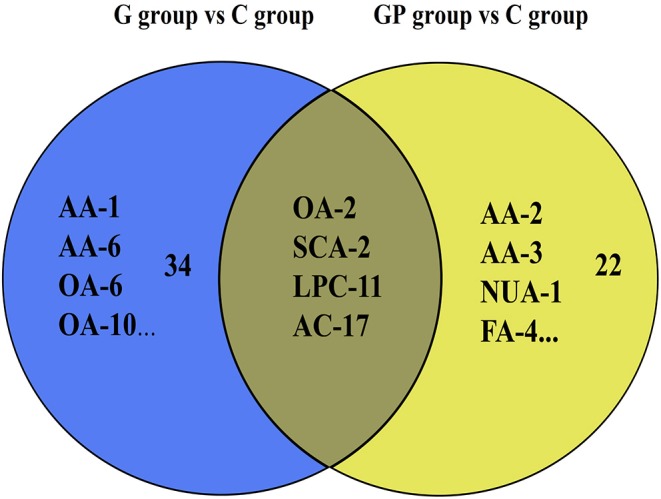
Venn diagram of the common discriminating metabolites identified between GEM-S and GEM-R groups both with GEM monotherapy and GEM combination. The blue ovals illustrate the differential metabolites with GEM alone compared with saline between GEM-S and GEM-R subgroup. The yellow ovals illustrate the differential metabolites with GEM plus nab-PTX compared with saline between GEM-S and GEM-R subgroup.

**Figure 5:**
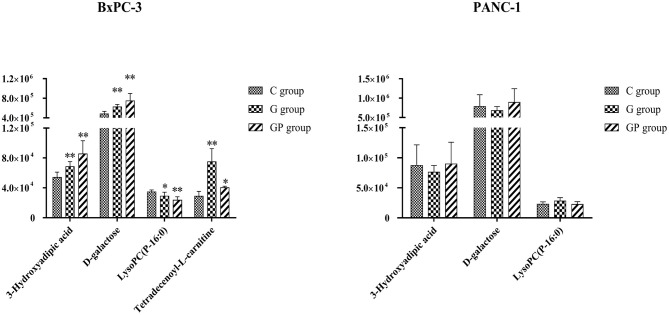
The relative amount changes of the common discriminating metabolites identified between GEM-S and GEM-R groups both with GEM monotherapy and GEM combination. **Significant difference (*P* < 0.01) compared with C group; *significant difference (*P* < 0.05) compared with C group. G, GEM; GP, GEM plus nab-PTX; C, untreated.

To better predict the efficacy of GEM-based treatment regimens, the common differential metabolites in serum including the differential metabolites from GEM-R and GEM-S tumor-bearing mice treated with GEM monotherapy or combination therapy were compiled. There were four common discriminating metabolites in the GEM-S and GEM-R subgroups treated with GEM monotherapy or combination therapy: 3-hydroxyadipic acid (OA-2), d-galactose (Sca-2), lysophosphatidylcholine [LysoPC (P-16:0), LPC-11], and tetradecenoyl-l-carnitine (AC-17) (Figure 4). The relative amounts of change in the common discriminating metabolites are shown in Figure 5; these four metabolites changed consistently and significantly in the GEM-S subgroup compared with the C group, regardless of treatment with GEM monotherapy or combination therapy. Compared to the C group, both the G and GP groups showed a significant increase in OA-2, Sca-2, and AC-17, and an obvious decrease in LPC-11. However, except for the identification of AC-17, OA-2, Sca-2, and LPC-11 in serum from the GEM-R subgroup, no differential metabolites were identified in the other treatment groups. In summary, these four metabolites showed significant differences in serum from the GEM-S and GEM-R PC models; thus, they might effectively predict the efficacy of chemotherapy in the context of both GEM alone and GEM plus nab-PTX.

## Discussion

Although previous studies have shown that ~70–90% of PC patients have KRAS mutation (34, 35), there are currently no effective therapeutics targeting KRAS. Haas et al. reported no significant difference in overall survival for KRAS wild-type vs. mutant patients, and KRAS mutation status is predictive rather than prognostic in advanced PC (34). Efforts focused on targeting PC have been disappointing (7, 36), so the standard of care for PC continues to be chemotherapy. However, chemoresistance is a major challenge in the treatment of PC (14, 15, 17, 18). The efficacy of GEM monotherapy for PC is limited by emerging drug resistance, which can be intrinsic or acquired after multiple treatment cycles. To overcome drug resistance, two regimens, including GEM plus nab-PTX, are usually applied, as first-line therapy for PC patients (37). However, not all patients benefit from this intense therapy, and clinicians lack predictive markers to help choose which patients will benefit or to predict when chemoresistance will occur.

In this study, we conducted a preliminary exploration of predictive indicators of the efficacy of GEM-based chemotherapy. We established GEM-S and GEM-R PC xenograft models and then analyzed the changes in metabolic profiles after treatment with GEM monotherapy and combination therapy to explore potential biomarkers for predicting the effect of GEM-based therapy. Considering that nab-PTX monotherapy is not used to treat PC in the clinic, the impact of nab-PTX alone on metabolism was not investigated. The tumor growth experiments showed that GEM monotherapy significantly inhibited tumor growth in the GEM-S subgroup but not in the GEM-R subgroup. However, GEM plus nab-PTX doublet therapy significantly inhibited tumor growth in both subgroups. These results were similar to clinical trial results (37, 38).

To better understand the effect of GEM-based chemotherapy on metabolism, we performed a metabolomics analysis of serum, liver, and tumor samples from GEM-S/R tumor-bearing mice treated with chemotherapeutics. The PLS-DA results showed that monotherapy and combination therapy had significant impacts on serum, liver and tumor metabolism (Figure 2). However, cluster analysis of these differential substances from different tissues showed that only serum differential metabolites in the GEM-S subgroup could significantly distinguish the drug regimens; these metabolites could not be separate in the other groups. We also obtained similar results when identifying differential metabolites in different groups (Supplemental Figures 3, 4). In the GEM monotherapy group, 39 differential metabolites in serum and five in the liver were identified in GEM-S tumor-bearing mice, but no differential metabolites were discovered in the tumor. On the other hand, one, seven, and one differential metabolites were identified in serum, liver, and tumor samples from GEM-R tumor-bearing mice, respectively. Furthermore, in the GEM plus nab-PTX group, 21, 3, and 2 differential metabolites were identified in serum, liver, and tumor samples from GEM-S subgroup, and 5, 2, and 2 differential metabolites were identified in serum, liver, and tumor samples from GEM-R subgroup. Based on the above results, we concluded that regardless of treatment with GEM monotherapy or combination therapy, the metabolic profiles in serum were more significantly different than those in the liver and tumor and thus could better reflect the effect of drugs on metabolism. It is well-known that changes in circulating metabolites associated with tumors might reflect alterations in metabolism within the tumor as well as general alterations in the host (39). In this study, we found that compared to control group, GEM-based chemotherapy had less effect on tumor and liver metabolites than on serum metabolites. Moreover, in the GEM-S group, both monotherapy and combination therapy led to significant metabolic changes, with 38 and 26 differential metabolites, respectively. Therefore, there is extensive clinical guiding significance through monitoring changes in serum metabolites after chemotherapy and exploring the differential metabolites in serum as biomarkers to predict the efficacy of chemotherapy.

In the clinic, the choice of monotherapy or combination therapy regimens for patients with advanced PC was based on the general state of the patients (5, 6). The clinical effectiveness of a treatment regimen in cancer patients is estimated by the Response Evaluation Criteria in Solid Tumors, at ~2 months after chemotherapy (40). If therapeutic effect could be predicted by monitoring serum metabolite changes after treatment, the treatment regimen could be adjusted as soon as possible thereby avoiding disease progression due to drug resistance and ensuring that PC patients receive timely and effective treatment. We identified four common differential metabolites in the serum of tumor-bearing mice treated with GEM alone or GEM plus nab-PTX. Compared with GEM-R mice, GEM-S mice showed a significant increase in OA-2, Sca-2, and AC-17 and a significant decrease in LPC-11 on GEM-based chemotherapy compared with the control (no treatment) (Figure 5). These metabolites have potential as molecular markers for discriminating bearing mice between GEM-S and GEM-R pancreatic tumors. However, we also found that the relative amount of OA-2 was higher in the GEM-R group than that in the GEM-S group in the absence of treatment. Therefore, we believe that the changes in these four differential metabolites in serum before and after treatment are more instructive for the clinical prediction of GEM-based treatment effects, and monitoring differential serum metabolites is expected to be useful in the clinic since serum is easy to obtain.

It is well-known that cancer cells can reprogram their metabolism to satisfy energy requirements and to preserve their integrity in harsh and hypoxic environments. Sac-2 is an energy source and also a necessary basic substrate for the biosynthesis of many macromolecules in the body. Sac-2 is involved in the biosynthesis of nucleotide sugars, which are the primary substrates for deoxyribonucleotide and ribonucleotide synthesis. GEM is a specific analog of the native pyrimidine nucleotide deoxycytidine and inhibits DNA synthesis through incorporation into DNA and inhibition of the enzyme ribonucleotide reductase (41). Furthermore, deoxyribonucleotide and ribonucleotide pools, which are both essential for DNA repair, are depleted by phosphorylated gemcitabine (41). In our study, Sac-2 levels increased significantly in GEM-S group after GEM-based chemotherapy, compared with no treatment, which may be related to the GEM-mediated inhibition of DNA synthesis. In addition, Sac-2 participates in the degradation of galactose, which can be transformed into α-d-glucose-6P, a component of glycolysis. The increase in Sac-2 may also be related to the inhibition of glycolysis by GEM-based chemotherapy.

With the exception of the Warburg effect, one of the most important metabolic aberrations in cancer cells is the elevated synthesis of lipids, which are building blocks for cell membrane formation during cell proliferation and signal transduction (42–46). LPCs were recognized as carriers of fatty acids, phosphatidylglycerol and choline between tissues (47) and were closely related to the occurrence and development of PC (47–50). Our results showed that the relative amount of LPC-11 in the GEM-S subgroup decreased markedly upon treatment with either GEM monotherapy or combination therapy compared with no treatment; LPC-11 could be a metabolic predictor of the efficacy of GEM-based chemotherapy. The carnitine system is another pivotal mediator in cancer metabolic plasticity, which is involved in the bi-directional transport of acyl moieties from the cytosol to the mitochondria and vice versa and thus plays a fundamental role in tuning the switch between the glucose and fatty acid metabolism (51). AC-17 is a long-chain acylcarnitine, which is transformed to long-chain acetyl-CoA, which participates in the β-oxidation of fatty acids, a process that provides energy for cancer cells. In this study, the relative amount of AC-17 increased significantly in GEM-S mice, especially in those treated with GEM alone, suggesting that GEM-based treatment may inhibit fatty acid β-oxidation to decrease the energy available to cancer cells. OA-2 is a dicarboxylic acid derived from the omega-oxidation of 3-hydroxy fatty acids. The relative amount of OA-2 increased significantly in GEM-S mice treated with GEM monotherapy and combination monotherapy. Although these metabolites have not been previously reported in the context of GEM-based PC treatment, they are theoretically worthy of further attention. Changes in these four metabolites may predict the efficacy of GEM-based treatment and be related to lipid metabolism; these hypotheses require further verification.

This study preliminarily explored predictive metabolic indicators of the efficacy of GEM-based chemotherapy in subcutaneous PC xenografts. Further studies are needed to verify whether the alterations in the metabolites identified in this study are similar in PC patients on GEM-based chemotherapy and to explore the mechanism by which GEM-based regimens for PC affect metabolism. In combination with clinical practice, experimental research will be performed to investigate the effects of differential changes in metabolites at different times after treatment to find the best assessment time and to better predict the effectiveness of GEM-based chemotherapy.

## Conclusion

The relative amount of 3-hydroxyadipic acid, d-galactose, LysoPC (P-16:0), and tetradecenoyl-l-carnitine were significant different changed between GEM-S and GEM-R pancreatic carcinoma xenograft model groups, regardless of treatment with GEM alone or GEM plus nab-PTX. Monitoring the changes of metabolites may be a viable option for improving acquired resistance and preventing the acquisition of chemoresistance in PC.

## Data Availability Statement

The datasets generated for this study are available on request to the corresponding author.

## Ethics Statement

This research was approved by the Ethics of Animal Experiments Committee of Harbin Institutes of Technology.

## Author Contributions

HL conceived and designed the experiments. DW, XLi, XZ, FH, XLu, LL, JZ, MD, and HY performed the experiments and analyzed the data. FH and DW wrote the paper. All authors read and approved the final manuscript.

### Conflict of Interest

The authors declare that the research was conducted in the absence of any commercial or financial relationships that could be construed as a potential conflict of interest.
