# Supplementary material for: Pharmacometabolomics Identifies 3-Hydroxyadipic Acid, d-Galactose, Lysophosphatidylcholine (P-16:0), and Tetradecenoyl-l-Carnitine as Potential Predictive Indicators of Gemcitabine Efficacy in Pancreatic Cancer Patients
Source: Front Oncol. 2020 Jan 29;9:1524. doi: 10.3389/fonc.2019.01524 (PMC7000527; doi:10.3389/fonc.2019.01524)
Supplement: Supplementary file 1 [file Data_Sheet_1.pdf]

Supplementary Material

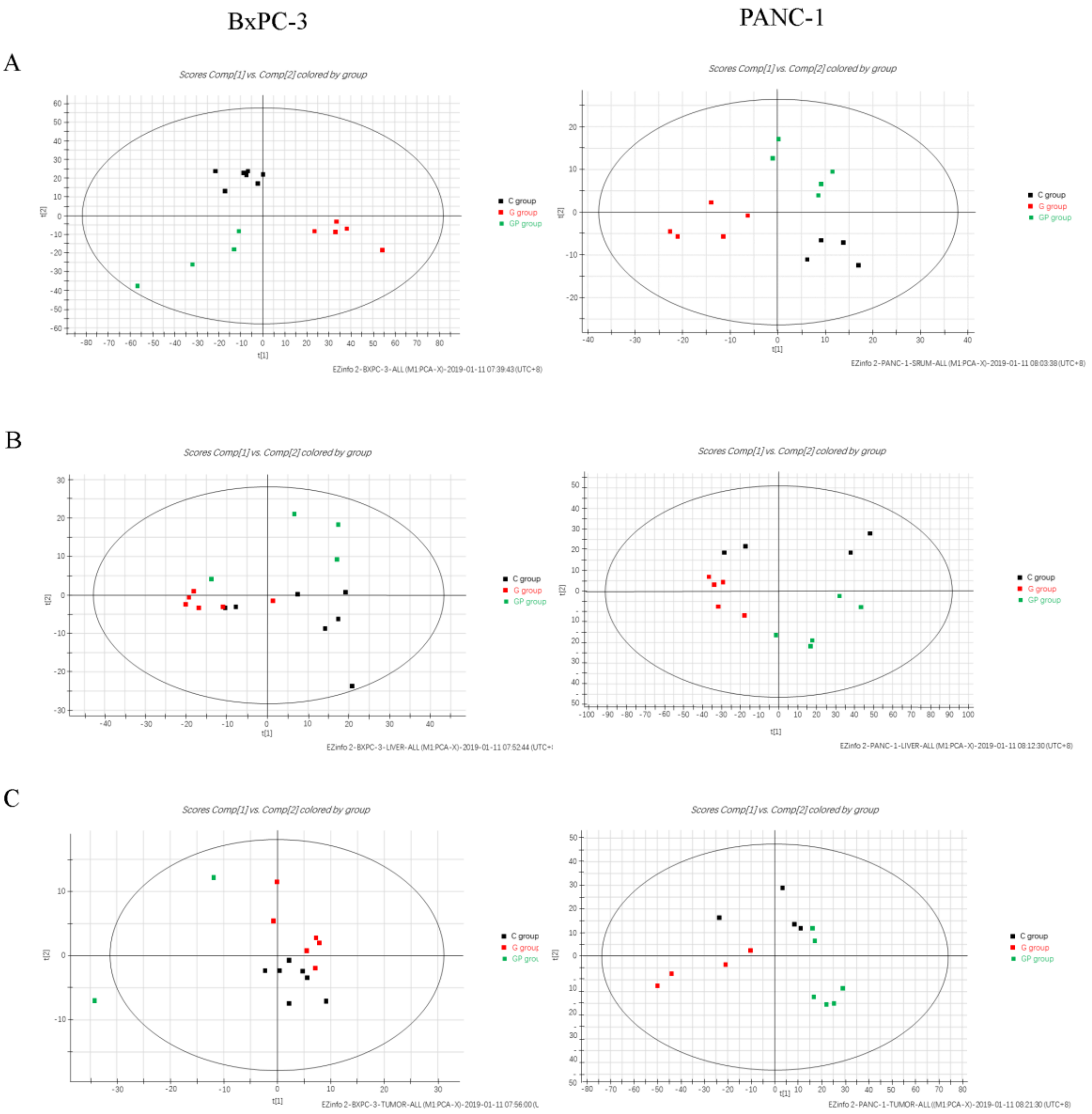

Supplemental Fig.1. The two-dimensional score plots of PCA of serum， liver and tumor samples to discriminate among GEM plus nab-PTX, GEM alone and untreated therapy in BxPC-3 and PANC-1 pancreatic tumor bearing mice.

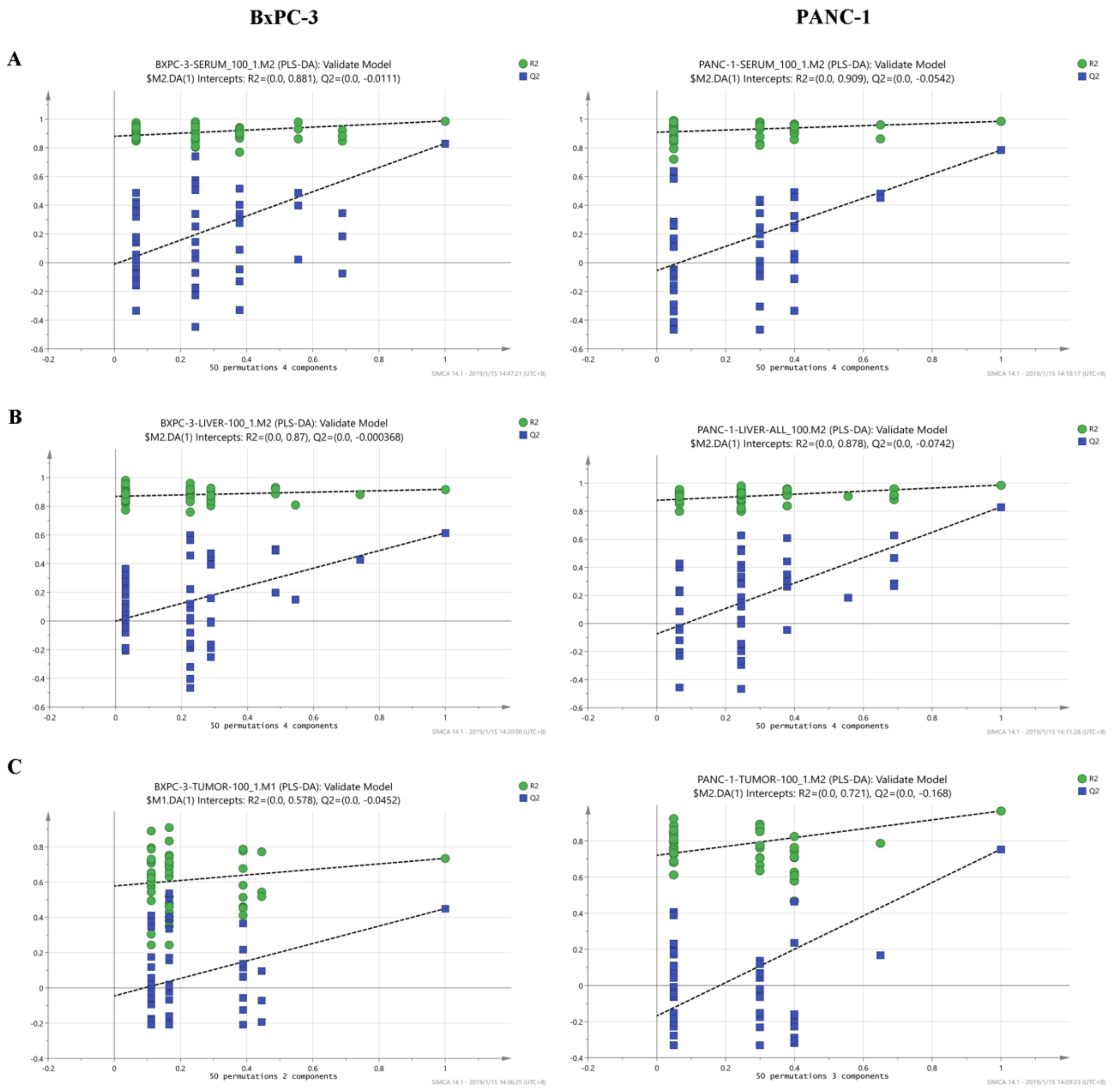

Supplemental Fig.2. The results of Cross Validation in serum, liver and tumor samples to discriminate among GEM plus nab-PTX, GEM alone and untreated therapy in BxPC-3 and PANC-1 pancreatic tumor bearing mice.

A

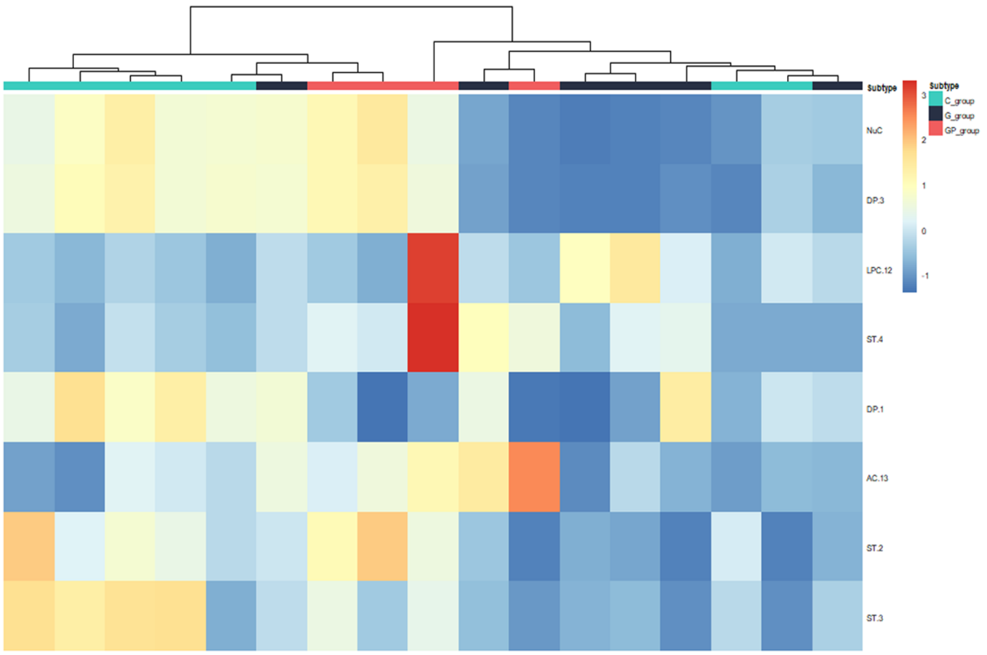
Subtype
NuC
DP.3
LPC.12
ST.4
DP.1
AC.13
ST.2
ST.3
3
2
1
0
-1
Subtype
C_group
G_group
GP_group

B

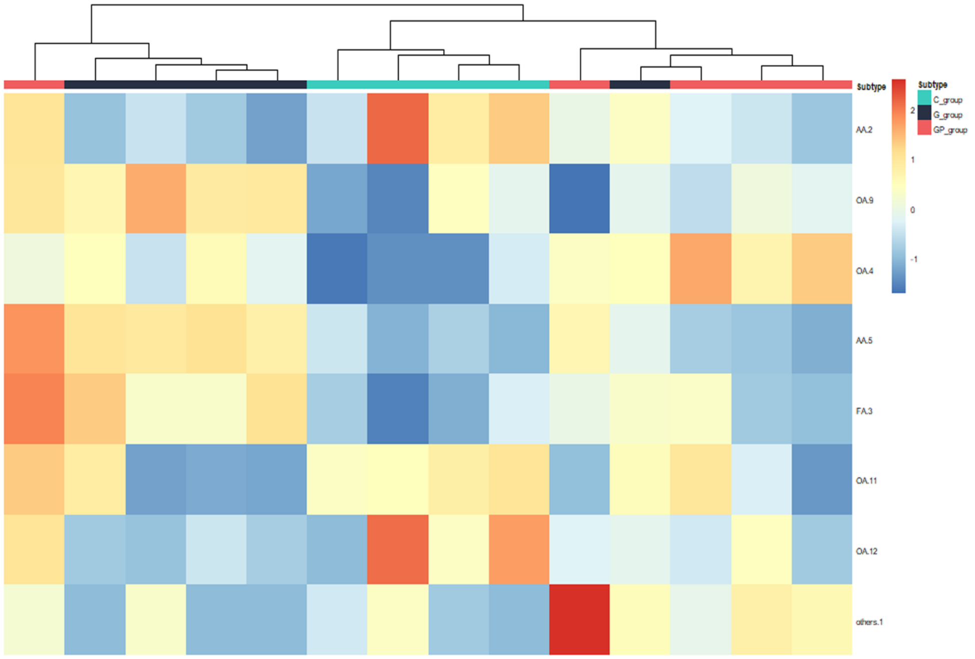
Subtype
AA.2
OA.9
OA.4
AA.5
FA.3
OA.11
OA.12
others.1
2
1
0
-1
Subtype
C_group
G_group
GP_group

Supplemental Fig.3. HCA-heatmap of the differential metabolites in the liver samples treated with GEM plus nab-PTX or GEM in GEM-S subgroup (A) and GEM-R subgroup (B).

A

B

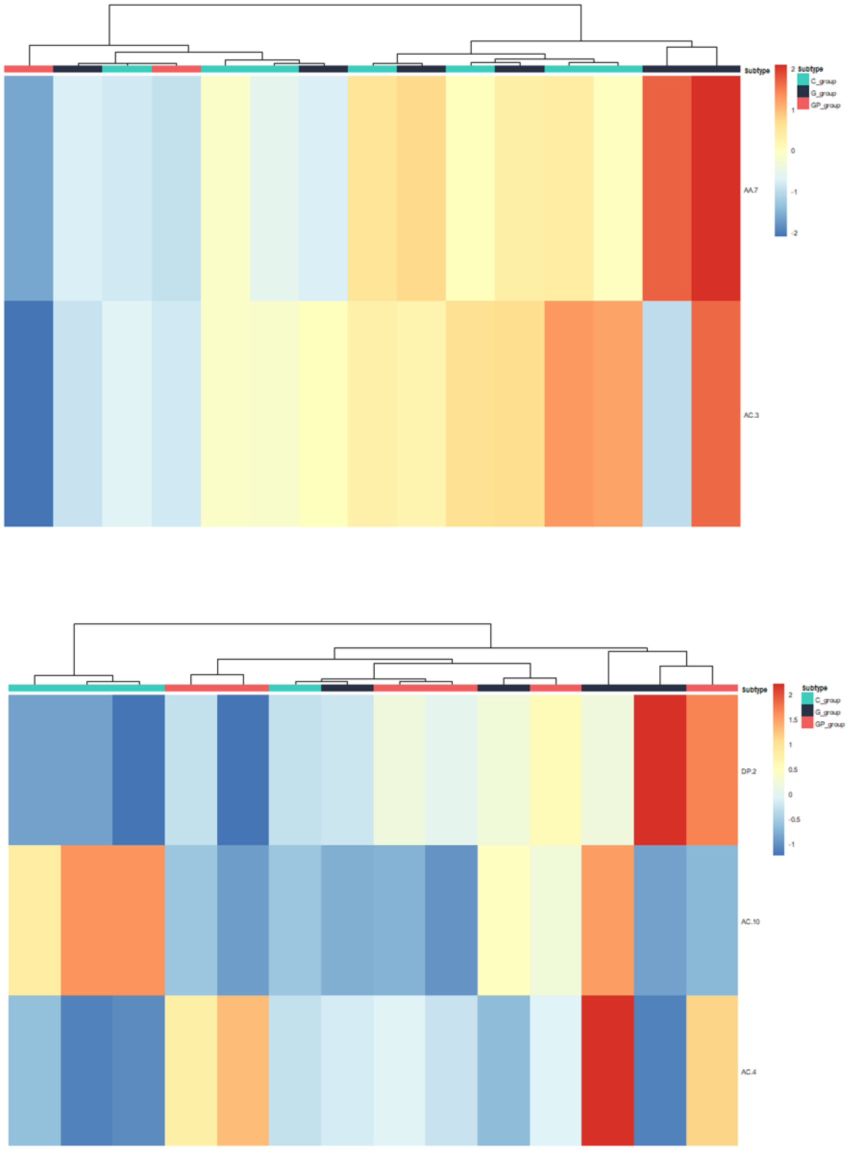

Supplemental Fig.4. HCA-heatmap of the differential metabolites in the tumor samples treated with GEM plus nab-PTX or GEM in GEM-S subgroup (A) and GEM-R subgroup (B).
