# Supplementary material for: Pharmacometabolomics Identifies 3-Hydroxyadipic Acid, d-Galactose, Lysophosphatidylcholine (P-16:0), and Tetradecenoyl-l-Carnitine as Potential Predictive Indicators of Gemcitabine Efficacy in Pancreatic Cancer Patients
Source: Front Oncol. 2020 Jan 29;9:1524. doi: 10.3389/fonc.2019.01524 (PMC7000527; doi:10.3389/fonc.2019.01524)
Supplement: Supplementary file 2 [file Data_Sheet_2.pdf]

frontiers

Supplementary Material

Supplemental Table 1. Details of 12 significant differential metabolites in liver treated with GEM alone.

Name	Group	Molecular formula	Ion（m/z）	RT/ min	ESI mode	Cells	Relative amount		Tendency
							C group	G group	G/C
L-Glutamine	AA-2	C5H10N2O3	145.0655	2.02	M-H	PANC-1	115252.4 ± 37532.97	53241.95 ± 22251.11	↓*
L-Phenylalamine	AA-5	C9H11NO2	164.0715	3.45	M-H	PANC-1	2050982 ± 113620.7	2730062 ± 200825.9	↑*
Histidinyl-Cysteine	DP-3	C9H14N4O3S	259.0889	1.93	M+H	BxPC-3	145497.6 ± 58453.46	62699.17 ± 49421.08	↓*
(R)-3-Hydroxy-hexadecanoic acid	FA-3	C16H32O3	271.2264	8.19	M-H	PANC-1	38514.01 ± 12488.95	81863.59 ± 12014.74	↑*
PC(O-16:0/0:0)	LPC-12	C23H48NO7P	482.3235	10.87	M+H	BxPC-3	7329.704 ± 6880.131	28526.68 ± 16538.42	↑*
Uridine	NuC	C9H12N2O6	245.0732	1.93	M+H	BxPC-3	605353 ± 255130	243855.4 ± 237920	↓*
Citric acid	OA-4	C6H8O7	191.0193	2.17	M-H	PANC-1	209280.5 ± 179406.9	700073.5 ± 151183.2	↑*
L-Glutamate	OA-9	C5H9NO4	146.0462	2.05	M-H	PANC-1	306643.7 ± 136167.4	551534.6 ± 96649.28	↑*

Pyrroline hydroxycarboxylic acid	OA-11	C5H7NO3	130.0505	1.57	M+H	PANC-1	337462.4 ± 41880.54	154772.4 ± 152234.8	↓*
Uric acid	OA-12	C5H4N4O3	169.0353	2.39	M+H	PANC-1	57260.21 ± 31833.62	20449.87 ± 7603.755	↓*
7α-Hydroxycholest-4-en-3-one	ST-2	C27H44O2	445.3314	11.80	M+FA-H	BxPC-3	71358.55 ± 42354.6	26134.49 ± 17366.96	↓*
Chenodeoxycholate	ST-3	C24H40O4	391.2832	6.80	M-H	BxPC-3	22894.33 ± 15435.9	6842.795 ± 3978.526	↓*

Details of the 12 significant differential metabolites in liver after treatment with GEM alone, compared with no treatment. Relative amount in LC-MS were represented as mean values ± standard deviation. * Significant difference (P<0.05) compared with C group; G, GEM; C, untreated.

Supplemental Table 2. Details of 5 significant differential metabolites in liver treated with GEM plus nab-PTX.

Name	Group	Molecular formula	Ion（m/z）	RT/ min	ESI mode	Cells	Relative amount						Tendency
							C group			GP group			GP/C
Octadecanoyl-L-carnitine	AC-13	C25H49NO4	428.3724	9.17	M+H	BxPC-3	68663.63	±	51990.76	238339.1	±	113066.6	↑ *
(Iso)leucyl-Phenylalanine	DP-1	C15H22N2O3	279.1746	2.11	M+H	BxPC-3	69684.77	±	26512.48	14384.21	±	29700.66	↓ *
Citric acid	OA-4	C6H8O7	191.0193	2.17	M-H	PANC-1	209280.5	±	179406.9	932068.5	±	207073	↑ *
Glycocholate	ST-4	C26H43NO6	466.3166	10.96	M+H	BxPC-3	13972.62	±	13253.58	94191.03	±	51220.09	↑ *
(10E)-9-Oxo-10-hexadecenoic acid	others-1	C16H28O3	269.2111	8.01	M+H	PANC-1	6251.903	±	6268.563	21252.29	±	11137.16	↑ *

Details of the 5 significant differential metabolites in liver after treatment with GEM plus nab-PTX, compared with no treatment. Relative amount in LC-MS were represented as mean values ± standard deviation. * Significant difference (P<0.05) compared with C group; GEM plus nab-PTX; C, untreated.

Supplemental Table 3: Details of 1 significant differential metabolite in tumor treated with GEM alone.

Name	Group	Molecular formula	Ion（m/z）	RT/ min	ESI mode	Cells	Relative amount		Tendency
							C group	G group	G/C
Glycyl-Phenylalanine	DP-2	$C_{11}H_{14}N_2O_3$	221.0916	3.59	M-H	PANC-1	2873.24 ± 1306.496	8348.628 ± 3607.585	↑*

Details of the 1 significant differential metabolites in tumor after treatment with GEM alone, compared with no treatment. Relative amount in LC-MS were represented as mean values ± standard deviation. * Significant difference (P<0.05) compared with C group; G, GEM; C, untreated.

Supplemental Table 4: Details of 4 significant differential metabolites in tumor treated with GEM plus nab-PTX.

Name	Group	Molecular formula	Ion（m/z）	RT/min	ESI mode	Cells	Relative amount						Tendency
							C group			GP group			GP/C
L-Tryptophan	AA-7	$C_{11}H_{12}N_2O_2$	205.0972	3.69	M+H	BxPC-3	2282055	±	672093.8	513711.9	±	256856	↓*
Decanoyl-L-carnitine	AC-3	$C_{17}H_{33}NO_4$	316.2473	5.58	M+H	BxPC-3	90949.07	±	24825.96	23472.12	±	11736.06	↓*
Decenoyl-L-carnitine	AC-4	$C_{17}H_{31}NO_4$	314.2314	5.46	M+H	PANC-1	10902.9	±	10699.21	50645.65	±	19791.61	↑*
Hexenoyl-L-carnitine	AC-10	$C_{13}H_{23}NO_4$	258.1699	5.29	M+H	PANC-1	39714.46	±	18998.65	7674.436	±	8771.906	↓*

Details of the 4 significant differential metabolites in tumo after treatment with GEM plus nab-PTX, compared with no treatment. Relative amount in LC-MS were represented as mean values ± standard deviation. * Significant difference ($P<0.05$) compared with C group; GP, GEM plus nab-PTX; C, untreated.
